# Repairing Hybrid Mg–Al–Mg Components Using Sustainable Cooling Systems

**DOI:** 10.3390/ma13020393

**Published:** 2020-01-15

**Authors:** David Blanco, Eva María Rubio, Marta María Marín, Joao Paulo Davim

**Affiliations:** 1 Department of Manufacturing Engineering, Industrial Engineering School, Universidad Nacional de Educación a Distancia (UNED), St/Juan del Rosal 12, E28040 Madrid, Spain; dblanco78@alumno.uned.es (D.B.); mmarin@ind.uned.es (M.M.M.); 2 Department of Mechanical Engineering, University of Aveiro, 3810-193 Aveiro, Portugal; pdavim@ua.pt

**Keywords:** hybrid components, light alloys, magnesium, aluminum, drilling, dry machining, cold compressed air, lubrication and cooling systems, arithmetical mean roughness, *Ra*, average maximum height, *Rz*, repair and maintenance operations

## Abstract

This paper focused on the maintenance or repair of holes made using hybrid Mg–Al–Mg components by drilling, using two sustainable cooling techniques (dry machining and cold compressed air) and taking surface roughness on the inside of the holes as the response variable. The novelty of the work is in proving that the repair operations of the multi-material components (magnesium–aluminum–magnesium) and the parts made of aluminum and magnesium (separately) but assembled to form a higher component can be done simultaneously, thus reducing the time and cost of the assembly and disassembly of this type of component. The study is based on a design of experiments (DOE) defined as a product of a full factorial 2^3^ and a block of two factors (3 × 2). Based on our findings, we propose that the analyzed operations are feasible under sustainable conditions and, in particular, under dry machining. Also, the results depend on the machining order.

## 1. Introduction

Today, energy efficiency and sustainability play an increasingly important role in the development of new materials and their applications, especially in the transport industry, due to the pollution generated by the vehicles or their different parts in many stages of their life cycle.

Therefore, in order to reduce such contamination, it is necessary to approach the problem in a global way, analyzing all the factors that can have an influence during the manufacturing, the use, the maintenance, the repair, and the recycling of each part.

Although new forms of energy are being investigated to propel vehicles, these new alternative energies need to be greatly improved to match the level of development achieved with fossil fuels. 

Until these new kinds of sustainable energy become competitive, in the transport sector, it is still essential to decrease the weight of vehicles to reduce the quantity of fossil fuels used, and, as a consequence, the pollution associated with its consumption. This is particularly true for the aeronautic and aerospace sectors.

To achieve this goal, research focuses on the use of the combination of different materials to create new multi-material components with better global properties than those of the original individual materials. These materials are called hybrid components, and the number of studies associated with them grew exponentially in recent years. Rubio and collaborators [1] affirmed that, among the possible combinations of materials used to form hybrid components with structural uses, the metal–polymer and metal–metal combinations are two of the most used, with aluminum alloys being most used to form these types of hybrid materials. Some of the main applications of these combination materials in the automotive sectors are car sleeper roofs, door structures, car fronts, tubular components of the exhaust system, and tubular components of the suspension system. In the aeronautic sector, the main applications are in the fuselage of airplanes and helicopters, the wings and rotors, and the other control surfaces (ailerons, flaps, spoilers, aerodynamic brakes, slats, and horizontal and vertical stabilizers).

The use of lightweight structural materials is widespread in various industries, particularly the aeronautics and automotive industries, since the weight of aircraft and cars is directly related to their consumption and pollution. A 10% reduction in the weight of an automobile can lead to improvements in fuel efficiency (by 8%), acceleration, and braking performance, and can reduce CO and hydrocarbon (HC) emissions by 4.5%, and NOx emissions by 8.8% [2]. In the civil aircraft industry, the weight of a Boeing 747–400 is approximately 183,500 kg, and the estimated fuel savings for an airplane is, over short distances, 117–134 kg of kerosene per kilogram of reduced weight and, over long distances, 172–212 kg of kerosene per kilogram of reduced weight [3].

Within the industries mentioned above, light alloys are used widely thanks to their excellent weight/mechanical strength ratio. Also, in recent years, combinations of light alloys (hybrid components) began to be used. Among them, it is possible to highlight the combination of aluminum and magnesium alloys. Both types of alloys can be combined with each other or with other lightweight and resistant materials to create hybrid materials, thereby extending the boundaries of the material’s property space [4,5,6,7,8]. Also, magnesium and aluminum present other interesting advantages in relation to sustainability; for example, both are easy to recycle [9,10]. 

Regarding magnesium, several recycling options exist. Firstly, when the scrap is of sufficient quality, it can be reprocessed to obtain parts with good specifications. Secondly, magnesium scrap can be used to produce other metallic materials. Finally, magnesium can be recycled for use as a raw material in the production of fertilizers [11]. 

Aluminum has 95% recyclability at the end of its useful life. Also, recycling saves 95% of the energy used in its initial production. Moreover, according to the Environmental Product Declarations (EPD), it was shown that aluminum contains an average of 39% recycled aluminum and 61% primary aluminum. In addition to its recyclability, aluminum has an excellent carbon footprint. The EPD states that, for its different types (anodized and lacquered, with and without a thermal break), the values of CO_2_ range between 10.3 and 11.8 kg of CO_2_ per kg of aluminum, and recycling can achieve exemptions of between 3.0 and 4.0 kg of CO_2_ per kg of aluminum.

The primary non-renewable energy used in the manufacture of aluminum products is another indicator of the impact they generate. As with the previous rate, considering all aluminum types included in the EPD, the primary energy is 420 MJ and 324 MJ per kg of aluminum. Therefore, aluminum recycling provides energy savings of between 49 and 38 MJ [9]. 

On the other hand, the geometric, dimensional shape and surface requirements are very strict in the aeronautics or aerospace sectors, which makes these parts types expensive and sometimes difficult or even impossible to keep stock of them ready for when it is necessary to maintain or repair damaged parts. Therefore, it is important to guarantee that it is possible to carry out efficient and sustainable repair or maintenance operations, thereby extending the lifetime of these parts and improving sustainability [12,13,14]. 

As the density of magnesium (1740 kg/m^3^) is the lowest among structural metals (being two-thirds that of aluminum and one-quarter that of steel), its alloys are interesting candidates for combination with heavier alloys to reduce weight. However, magnesium has low machinability and high flammability (especially as powder or chips). In fact, the magnesium flame temperature and its alloys can reach 3100 °C and, once the fire starts, it is difficult to extinguish since there is continuous combustion of nitrogen, carbon dioxide, and water [15]. Also, molten magnesium reacts violently with water. For these reasons, it is necessary to study the behavior of magnesium when it is mechanized along with other materials (forming hybrid components), testing if such combinations are suitable for manufacturing, repair, and maintenance. For example, steel produces sparks during machining at cutting speeds between 200 to 300 m/min [16], and this can be extremely dangerous if magnesium is present. 

Therefore, when magnesium-containing hybrid components are going to be mechanized, it is necessary to take certain security measures regarding the lubricants or coolant systems employed. Depending on the material or materials with which magnesium is combined, different lubrication/cooling techniques can be used [17,18] both individually (dry machining [19,20,21,22,23,24,25,26,27,28,29,30,31], minimum quantity lubrication (MQL) [30,31,32,33,34,35,36,37,38,39,40,41,42], solid lubrication [43,44,45,46,47], cryogenic cooling [48,49,50,51,52,53,54,55], gaseous cooling [56,57,58,59,60,61], nanofluids [62,63,64,65,66], and sustainable cutting fluids [67,68,69,70,71,72]) and in combination [73,74,75,76]. Some of these techniques were tested in several previous works with the intention of better describing the behavior of the individual materials (especially aluminum [19,20,21,22,23], titanium [30,31,39], and magnesium [16,28,77,78,79,80,81,82,83,84,85,86,87,88,89,90,91,92,93,94,95,96,97,98,99,100,101,102,103,104]). From the point of view of the optimization of the costs of the process and its sustainability, the ideal would be (1) to be able to completely eliminate lubricants or coolants and carry out dry machining, (2) to test more recently developed techniques (such as machining with minimum quantity of lubricant, cold compressed air or cryogenic refrigerants), and (3) to develop new lubricants or refrigerants compatible with magnesium.

The machining of this type of hybrid component results in an increase in process instability due to the different properties of the materials that form them and their particular cutting characteristics [12]. This makes it necessary to determine the best cutting parameters for each combination of materials, especially when strict design requirements must be reached. Although the literature contains an important number of experimental works with regard the machining of hybrid components, only some of them addressed hybrid components based on magnesium. Most studies focused on machining processes [105,106,107,108,109,110,111,112,113] and tried to find the optimal combination of machining conditions and lubrication/cooling systems by means of experimental tests, taking the surface roughness required in a particular industry sector or application as a response variable. Others dealt with friction and wear between contact materials [114,115], the effects of pre-treatments on the adhesion of hybrid materials [116], innovative techniques for forming these types of components [117,118], or identification of some of the most prominent issues.

The scope of this work is to prove that repair and maintenance operations can fix holes made in pieces of hybrid components based on magnesium and aluminum, not only in an efficient way, but also sustainably. Therefore, considering the above, this experimental study focused on the drilling of hybrid Mg–Al–Mg components. For decades, aluminum and magnesium were used (separately) in the aeronautical sector due to their good weight/mechanical properties. Thus, given that the density of magnesium is two-thirds that of aluminum, it was hypothesized that they could be used together, reducing weight by replacing aluminum with magnesium where possible.

The drilling process is used regularly in this sector and, thus, it was well analyzed; for only the assembly of the wing to the fuselage, thousands of holes are required [119,120]. 

The repair and maintenance operations were selected as they represent an additional challenge versus those of manufacturing, since, as previously mentioned, the lack of parts in aeronautical stock means having to do the repair in the shortest possible time to reduce the costs associated with the downtime of the aircraft. Also, two cooling systems (dry machining and cold compressed air) were tested to analyze the sustainability of the process. Surface roughness was chosen as a response variable as it is one of the most widespread in the literature, thereby allowing a better contrast of the results obtained; the required values for the sector are also standardized (0.8 µm < *Ra* < 1.6 µm) [121]. 

The novelty of the work is in proving that the repair operations of multi-material components (magnesium–aluminum–magnesium) and the parts made of aluminum and magnesium (separately) but assembled to form a higher component can be repaired simultaneously. This approach saves time and reduces cost.

## 2. Methodology

As this work is part of a broader research project that involves different geometries, material combinations, cutting conditions, tools, and lubrication/cooling systems, the methodology is similar to that followed in other previous works [105,106,107,108] and is based on the guidelines given by Montgomery [122].

### 2.1. Pre-Experimental Planning

Here, we report the findings of an experimental study addressing the repair or maintenance of holes made in parts of Mg–Al–Mg hybrid components using sustainable cooling systems. The study focuses on the aeronautical sector. As the response variable, we chose surface roughness since it is commonly used as a reference of quality in aeronautical components and is also used to evaluate the efficiency of the machining processes; thus, there are several works in the literature with which to make comparisons. 

To determine the factors, levels, and range of their values, it should be noted that these are repair operations; hence, the depth of cut must be as small as possible to maintain the dimensional design requirements. On the other hand, since these are two non-ferrous alloys with similar machinability characteristics, it would be sufficient to test a unique type of tool. Moreover, since two cooling systems (dry machining and cold compressed air) were tested, and only a single pre-drilled part with eight holes through the Mg–Al–Mg combination was available, it was necessary to adapt the remaining factors and levels to the number of holes. Therefore, we decided to use the feed rate and the spindle speed (and two levels for each) as factors.

Additionally, as it was thought that the surface of the drilled holes could be damaged, not only by the cutting process but also due to friction caused by the chips inside of them (due to the accumulation along the mechanized length), and seeing that a similar factor was taken into account in other works [19,20,21,22,23,24,25,26], it was decided to include two additional factors related to the location relative to the insert (with three levels, one for each one of the stacks: Mg–Al–Mg) and related to the location relative to the specimen, that is, each hole (with two levels, one at the entry of the holes and one at the exit holes), where measurement of the surface roughness was taken.

### 2.2. Experimental Design

Considering everything explained in the pre-experimental planning, the depth of cut and the type of tool do not affect the design of experiments (DOE) since they only have one level. For the feed rate, *f* (mm/rev), the spindle speed, *N* (rpm), and the type of cooling system, *C*, two levels were taken for each, i.e., (*f1*, *f2*), (*N1*, *N2*), and (*C1*, *C2*), respectively. In the same way, for the additional factors where the surface roughness was measured, i.e., location relative to the insert, *LRI*, and location relative to the specimen, *LRS*, three (*LRI1*, *LRI2*, *LRI3*) and two (*LRS1*, *LRS2*) levels were taken, respectively. Table 1 describes the factors and levels selected for this experimental analysis. 

The surface roughness was taken as the response variable, and the average roughness values (*Ra*) and the average maximum height (*Rz*) were measured in the different zones defined by the factors location relative to the insert (*LRI*) and location relative to the specimen (*LRS*). The factors and levels are shown in Table 1, and a DOE, as a product of a full factorial 2^3^ and a block of two factors (3 × 2), was defined with a total of eight experimental re-drills and 24 measurements of the surface roughness, which provided a total of 48 values (24 of *Ra* and 24 of *Rz*). Also, the design was randomized to reduce the influence of non-considered variables [122] (Table 2). 

### 2.3. Performing the Experiment

Before carrying out the re-drilling tests, it was necessary to collect the specimens of the hybrid parts, the tools, and the cooling systems, as well as introduce the parameter values into the machine tools and establish cutting conditions and data collecting protocols. Next, the machining operations were carried out and, finally, photographs and videos of the trials were taken for subsequent analysis.

### 2.4. Statistical Analysis of the Data 

Once the machining process was finished, the arithmetical mean roughness (*Ra*) and average maximum height (*Rz*) were measured. The data were statistically analyzed, including an analysis of variance (ANOVA) to identify the influential factors for surface roughness variation and the interactions among them.

### 2.5. Conclusions

The main conclusions extracted from the descriptive analysis of the obtained results and their statistical analysis were established.

## 3. Applications and Results

### 3.1. Materials

The hybrid component specimen was made of three parallelepiped plates of dimensions 50 × 50 × 15 mm pre-drilled with eight holes of 8 mm in diameter. The three plates were mechanically fixed so that they could be easily disassembled to take the measurements inside the holes. The plates placed above and below were of magnesium alloy (UNS M11917), and the other (placed between them) was of aluminum alloy (UNS A92024). The chemical composition of both materials is given in Table 3. 

These materials were selected because the authors had previous experience in their machining, both independently [12,13,14,16,17,18,19,20,21,22,23,25,28,29,30,31,32,77,78,79,82,83,84,85,86,87,88,89,90,91,92,93,94] and together [105,106,107,108]. Also, there were interesting works of other researchers in the literature, thus allowing comparisons to be drawn [15,24,26,27,80,81,95,96,97,98,99,100,101,102,103,104].

### 3.2. Tools

As the target of this study was to analyze the feasibility of carrying out the repair and maintenance operations in an efficient and sustainable way, a single level for the depth of cut factor, *d*, was taken (*d* = 0.5 mm, using of a 9-mm-diameter drill bit). On the other hand, keeping the depth of cut at a low level also helps to keep the cutting temperature low and, therefore, to keep the magnesium temperature far from its ignition temperature.

**Figure 1:**
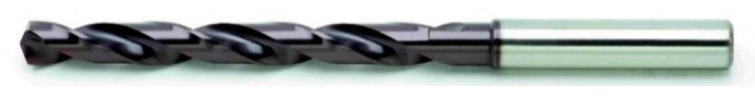
Helical drill bits HSS-E-PM A1 1257 manufactured by Garant [123].

The tools used in the trials were helical drill bits of high performance. They were made of a high-speed-steel (known as Cobalt Steel, HSSE, or HSS-E), obtained by powder metallurgy (PM) (Figure 1). The tools, with reference HSS-E-PM A1 1257, were purchased from Garant (Hoffmann Iberia, San Fernando de Henares, Madrid, Spain). 

Their dimensions were 9 mm of diameter, 81 mm of helical length, and 131 mm of total length. In addition, their special geometry allowed self-centering and optimal chip evacuation. 

### 3.3. Machines and Equipment

**Figure 2:**
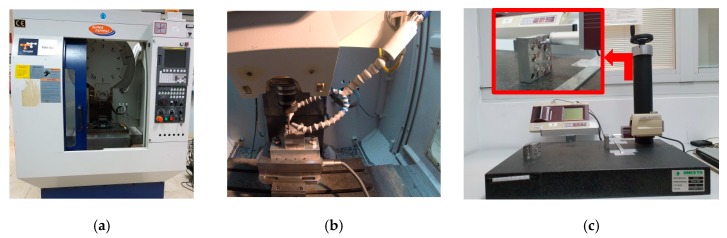
(**a**) Tongtai TMV510 machining center; (**b**) details of the Vortec Cold Air Gun during the trials; (**c**) Mitutoyo Surftest SJ 401 roughness tester.

The trials were carried out in a Tongtai TMV510 machining center (Tongai Machine &Tool Co., Luzhu Dist, Kaohsiung City, Taiwan) equipped with a Control Numeric Computer (CNC) Fanuc (Fanuc Iberia, Castelldefels, Barcelona, Spain) (Figure 2a). A drilling cycle was programmed that made the tool penetrate 10 mm, and then return to evacuate the generated chips. The same sequence was repeated until the tool crossed the entire width of the piece formed by the three stacks of Mg–Al–Mg. This was done so that the accumulated chips inside the holes did not scratch the surface or stop or hinder the tool inside the piece. Cold compressed air (CCA) was used as the cooling system, implementing a Vortec Cold Air Gun (Vortec, Cincinnati, Ohio, USA) (Figure 2b). The roughness measurements were taken using a Mitutoyo Surftest SJ 401 roughness tester (Figure 2c) with the following settings: measuring range, 800 µm; resolution, 0.000125 µm; transverse length, 25 mm; cut off, 0.8 mm; scan rate, 4 mm (*N* = 5); the standard ISO 1997 [124] was used. 

### 3.4. Experimental Tests

The design of experiments, the materials, tools, machines, and equipment used in the trials, and the parameter value ranges are given in Table 4.

**Figure 3:**
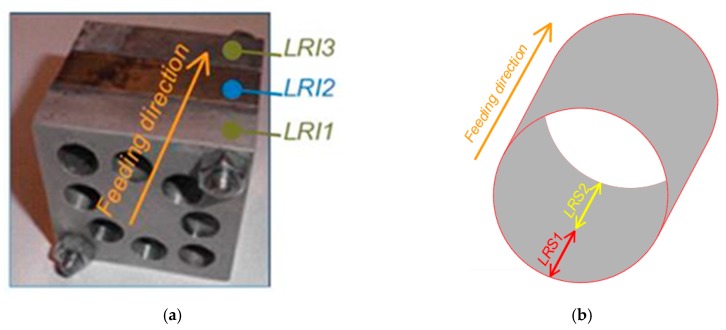
The concrete locations of the measurement zones of the surface roughness: (**a**) location relative to the insert, *LRI;* (**b**) location relative to the specimen, *LRS*.

**Figure 4:**
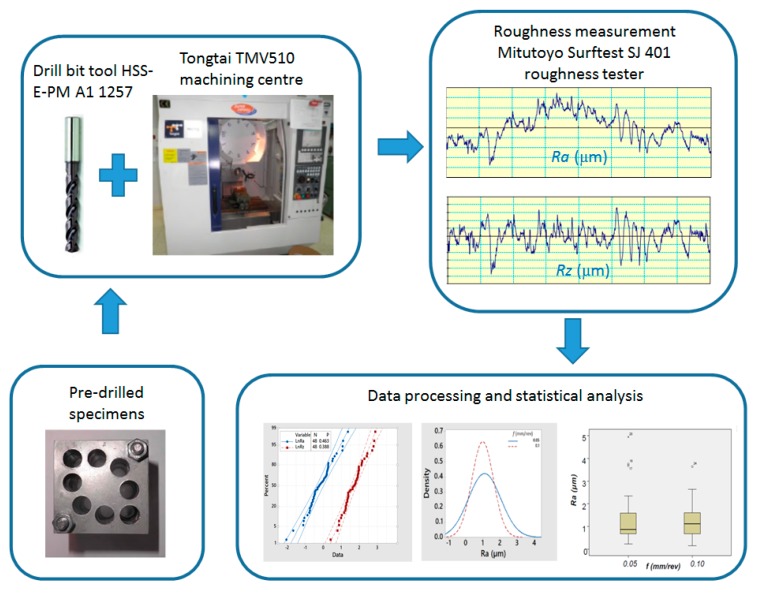
The experimental set-up.

The locations of the measurement zones of the surface roughness are shown in Figure 3. *LRI1* denotes the first magnesium plate, *LRI2* denotes the aluminum plate, and *LRI3* denotes the second (and last) magnesium plate. *LRS* took into account the location of the measuring zone inside each hole after re-drilling it, and its levels were defined as *LRS1* (the specimen entry zone) and *LRS2* (the specimen exit zone). Figure 4 provides a graphical summary of the experimental set-up.

### 3.5. Analysis and Discussion of the Results

After performing the eight re-drilling tests, surface roughness measurements were made in each hole in the three plates (entry and exit zones). The values of the arithmetical mean roughness (*Ra*) and the average maximum height (*Rz*) were calculated (in micrometers) and are given in Table 5. 

Initially, a descriptive method was used to analyze the *Ra* and *Rz* values. The obtained results are separated into Table 6 and Table 7. Table 6 and Table 7 give the values of *Ra* and *Rz*, respectively, in each plate (both in the entry and exit zones of the holes).

**Figure 5:**
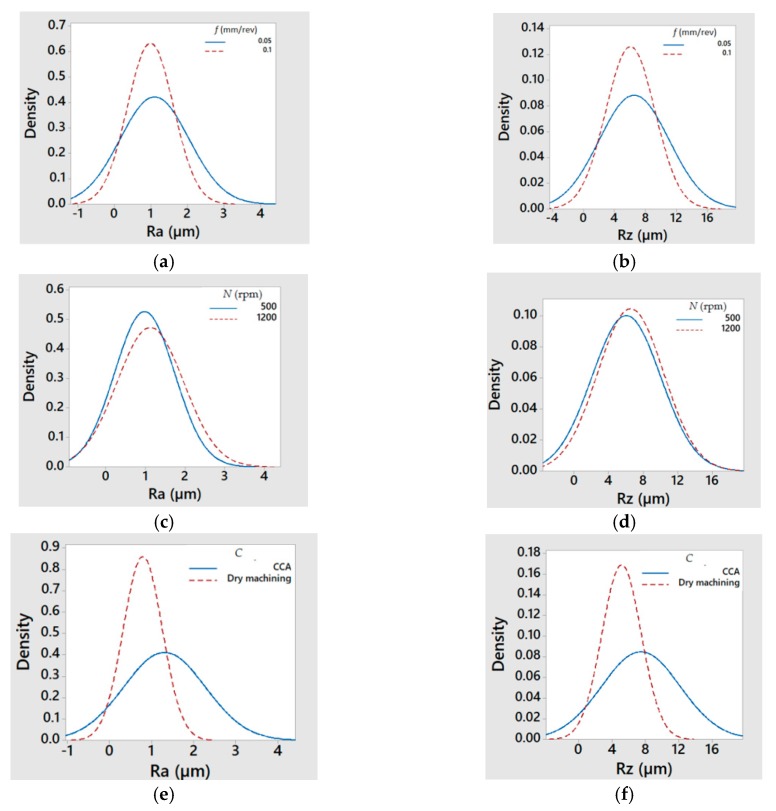

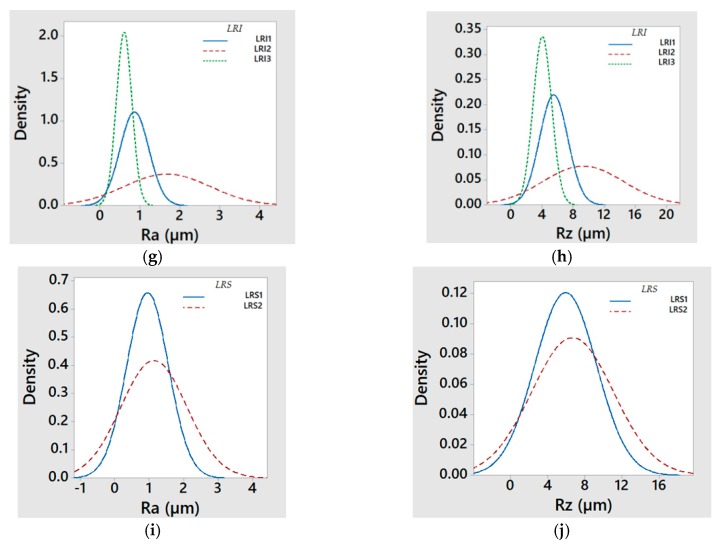
Normal distribution of *Ra* (µm) and *Rz* (µm), respectively, with respect to (**a**,**b**) feed rate, *f* (mm/rev); (**c**,**d**) spindle speed, *N* (rpm); (**e**,**f**) type of cooling system, *C*; (**g**,**h**) location regarding insert, *LRI*; (**i**,**j**) location regarding specimen, *LRS*.

From the *Ra* and *Rz* values given in Table 6 and Table 7, the graphics of Figure 5 were drawn. Figure 5 shows the normal distribution of *Ra* (left column) and *Rz* (right column) with respect to feed rate, *f* (mm/rev), (a) *Ra* and (b) *Rz*; spindle speed, *N* (rpm), (c) *Ra* and (d) *Rz*; type of cooling system, *C*, (e) *Ra* and (f) *Rz*; location relative to the insert, *LRI*, (g) *Ra* and (h) *Rz*; and location relative to the specimen, *LRS*, (i) *Ra* and (j) *Rz*.

Taking into account that a good behavior of the results is considered when the obtained values are concentrated in the interval [0.8 µm; 1.6 µm] given by the standard [121], a first approach to the analysis can be made by observing data collected in Table 6 and Table 7 and graphics from Figure 5. Thus, it was possible to affirm that *Ra* and *Rz* had a similar behavior for the cutting parameters; however, they were perhaps slightly better for high feed rates (*f* = 0.10 mm/rev) and dry machining and they were very similar for both tested values of the spindle speed (perhaps slightly better for low values *N* = 500 rpm). Regarding the location relative to the insert, in both magnesium plates, *Ra* and *Rz* were better than in the aluminum plate. Also, when comparing the results of the first and the last magnesium plates, the results were lower for the latter (*LRI3*). However, *LRI1* was considered as better since the surface roughness values were closer to the standard values used in the aeronautic sector (between 0.8 µm and 1.6 µm) [121]. Finally, regarding the location relative to the specimen, the results were lower at the entry of the holes than at the exit.

**Figure 6:**
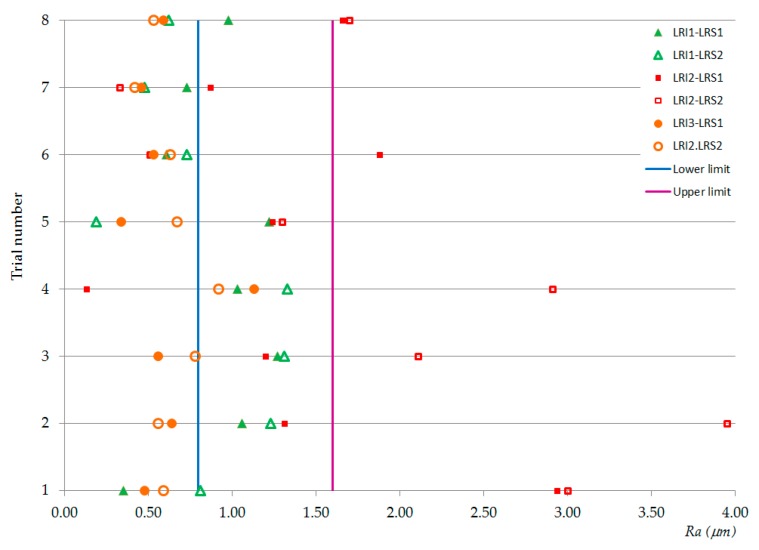
Graphic representation of the *Ra* values.

**Figure 7:**
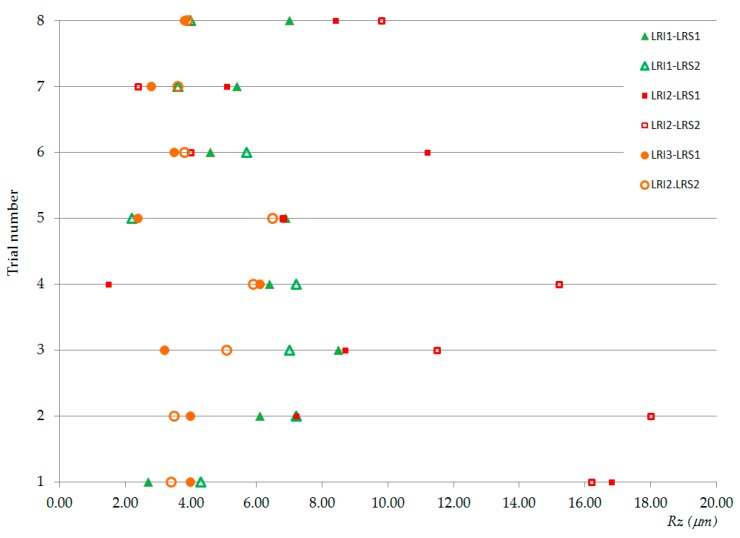
Graphic representation of the *Rz* values.

**Figure 8:**
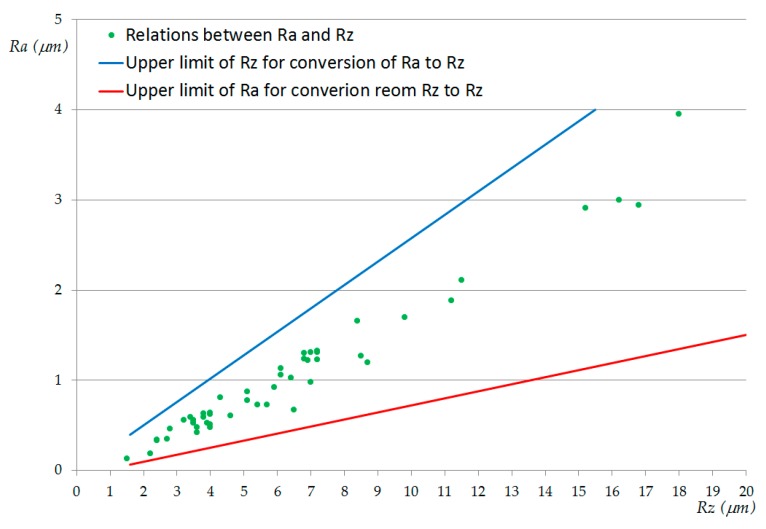
The relationship between *Ra* and *Rz* values.

The values from Table 6 and Table 7 are plotted in Figure 6 and Figure 7, respectively, revealing possible combinations of parameters that could be used for repairing hybrid parts by re-drilling. Figure 8 plots the *Ra* and *Rz* values obtained in the trials and collected in Table 5. All of them were inside of the usual upper and lower limits given in the chart of conversion relations between *Ra* and *Rz*, according to DIN 47 [125].

Observing the *Ra* and *Rz* values in Figure 6 and Figure 7, we see that, for the aluminum plate, the roughness values were higher than for the magnesium plate, especially at the exit of the holes and when using cold compressed air as the cooling system. Therefore, it seems reasonable to select aluminum as the more critical material when establishing the cutting parameters. As the results were better at the entry of the holes, it would perhaps be possible to improve the results by modifying the geometry of hybrid component, for example, by searching for the adequate proportions of the thicknesses of the combined materials. On the other hand, in the second plate of magnesium, most of the surface roughness values (except the obtained one for *f* = 0.05 mm/rev, *N* = 1200 rpm, and *C* = CCA) were lower than those established in the design requirement standards. Therefore, roughness values might be improved by drilling halfway, turning the hybrid component, and then continuing to drill from the opposite side.

Reviewing the surface roughness values at the entry of the aluminum holes (Table 6 column *LRI2*, *LRS1*), it can be seen that test numbers 2, 3, 5, and 7 presented values within the range of the values given by the standard (0.8 µm < *Ra* < 1.6 µm) [121]. Among them, test numbers 2 and 3 were within the same range for the first magnesium plate; test number 5 could be an option if the magnesium plates were about half as thick, since the *LRI1* had values within such an interval; test number 7 indicated room for improvement because the roughness value of the aluminum was close to the lower limit of the roughness required in the aeronautic sector (0.8 µm). In fact, comparing the roughness values obtained in test number 7 with those from test number 8, we propose that a new parameter combination is possible by selecting a spindle speed equal to 1000 rpm or near this value; this would also increase the feed speed, decrease the machining time, and, consequently, improve the efficiency of the process.

In addition, an ANOVA was performed to identify the factors that influence the variation of the response variables, *Ra* and *Rz*. To apply an ANOVA, it is necessary that the variables meet three conditions: (1) each data group must be independent, (2) the results obtained for each group must follow a normal distribution (although a breach of this assumption is supported when the distribution is symmetric), and (3) the variances of each data group must not differ significantly (homoscedasticity).

Using the data extracted directly from the experiment, the *Ra* and *Rz* values did not follow a normal distribution (Shapiro–Wilk test *p*-value < 0.05). Therefore, the data were processed using logarithmic transformation, maintaining its order but softening the effect of outliers. 

**Figure 9:**
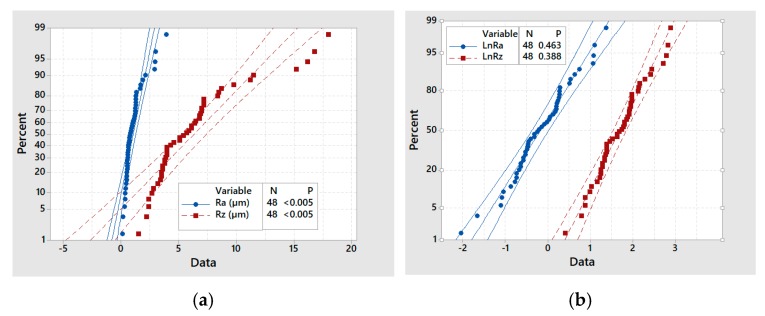
Probability plots: (**a**) *Ra* and Rz; (**b**) Ln*Ra* and Ln*Rz*.

By this approach, normally distributed Ln*Ra* and Ln*Rz* values (Shapiro–Wilk test *p*-value > 0.05) were obtained (Figure 9). In addition, the condition of homoscedasticity was also fulfilled (Levene statistic, *p*-value > 0.05), and independent data groups had a similar number of cases (Table 8). In the analysis, interactions up to the third order were considered, and successive iterations were performed until all values were significant. In each iteration, the statistically less significant effect was excluded if it had a *p*-value greater than 0.05. Table 9 and Table 10 give the outcome of the first and the last ANOVA over *LnRa*, and Table 11 and Table 12 collect the outcome of the first and the last ANOVA over *LnRz*, respectively.

Taking into account the results shown in Table 10 and Table 12, we conclude that the most influential factors, in both cases, were the location relative to the insert (*LRI*) and the type of cooling system (*C*), and that there were no interactions among factors with influence. 

Considering the re-drilling surface roughness variability of hybrid Mg–Al–Mg components explained by the statistically significant effect obtained from the ANOVA, the percentage of variability attributed to each factor is shown in Table 13, and the contribution of each effect was obtained as the percentage of the sum of squares values of each significant effect relative to the sum of squares of all significant effects.

## 4. Conclusions

This work focused on the maintenance and repair of holes made of hybrid Mg–Al–Mg components by drilling, using two sustainable cooling techniques: dry machining and cold compressed air. The aeronautic and aerospace sectors were selected as relevant applications. In such sectors, the pieces have strict design requirements for surface roughness (0.8 µm < *Ra* < 1.6 µm). *Ra* and *Rz* were taken as response variables. From our analyses, we propose that *Ra* and *Rz* values have similar behaviors and they exhibit the following characteristics:

▪ They are better for high feed rates and dry machining.
▪ They are very similar for both tested values of spindle speed, although perhaps slightly better for low values.
▪ They are, regarding the location relative to the insert, better in both magnesium plates than in the aluminum plate; also, when comparing the results of the first and the last magnesium plate, the results were lower for *LRI3*, yet the values for *LRI1* were considered better since the surface roughness values were closer to the aeronautic industry standard.
▪ They display, for the location regarding specimen, better behavior at the entry of the holes than at the exit.
▪ They are higher in the plate of aluminum than in the magnesium one, particularly at the exit of the holes and, in a more pronounced way, using cold compressed air as a cooling system. Therefore, aluminum is considered a more valuable material when selecting the cutting parameters. Also, as the results are better (lower values) at the entry of the holes than at the exit, it will perhaps be possible to improve the results by modifying the geometry of the hybrid component, for example, by searching for the adequate thicknesses among the different combined materials.
▪ They are lower, in most cases, in the second plate of magnesium than the established standard. Therefore, the process can be influenced by the drilling direction, and it could be improved by drilling halfway, turning the part, and drilling again from the opposite side.

In addition, from the ANOVA analysis, we found that the factors that influence the response variables *Ra* and *Rz* are location relative to the insert and type of cooling system, with percentages of influence of 72.6% and 27.4%, respectively, for Ra, and 79.2% and 20.8%, respectively, for *Rz*. 

With this work, we showed that it is possible to simultaneously repair magnesium–aluminum–magnesium multi-material components and parts made of aluminum and magnesium (separately) but assembled to form a higher component using sustainable cooling systems (dry machining). This approach reduces the time and cost associated with the assembly and disassembly of these types of components during maintenance or repair.

In conclusion, we propose three ways to optimize (or at least improve) the process: (1) using different parameters values (for example, higher values of the spindle speed that increase the efficiency of the process); (2) designing a hybrid component with new proportions of the thicknesses of the materials combined; (3) applying other drilling sequences (e.g., firstly drilling halfway and then turning the part and drilling from the opposite side).

## Figures and Tables

**Table 1:** Factors and levels.

Factors	Levels
Feed rate, *f* (mm/rev)	*f1*, *f2*
Spindle speed, *N* (rpm)	*N1*, *N2*
Type of cooling system, *C*	*C1*, *C2*
Location relative to the insert, *LRI*	*LRI1*, *LRI2*, *LRI3*
Location relative to the specimen, *LRS*	*LRS1*, *LRS2*

**Table 2:** Experimental design: product of a full factorial 2^3^ and a block of two factors (3 × 2).

No.	f *	N **	*C*	*LRI*	*LRS*	No	f *	N **	*C*	*LRI*	*LRS*	No.	f *	N **	*C*	*LRI*	*LRS*
1	*f1*	*N1*	*C1*	*LRI1*	*LRS1*	1	*f1*	*N1*	*C1*	*LRI2*	*LRS1*	1	*f1*	*N1*	*C1*	*LRI3*	*LRS1*
1	*f1*	*N1*	*C1*	*LRI1*	*LRS2*	1	*f1*	*N1*	*C1*	*LRI2*	*LRS2*	1	*f1*	*N1*	*C1*	*LRI3*	*LRS2*
2	*f1*	*N2*	*C1*	*LRI1*	*LRS1*	2	*f1*	*N2*	*C1*	*LRI2*	*LRS1*	2	*f1*	*N2*	*C1*	*LRI3*	*LRS1*
2	*f1*	*N2*	*C1*	*LRI1*	*LRS2*	2	*f1*	*N2*	*C1*	*LRI2*	*LRS2*	2	*f1*	*N2*	*C1*	*LRI3*	*LRS2*
3	*f2*	*N1*	*C1*	*LRI1*	*LRS1*	3	*f2*	*N1*	*C1*	*LRI2*	*LRS1*	3	*f2*	*N1*	*C1*	*LRI3*	*LRS1*
3	*f2*	*N1*	*C1*	*LRI1*	*LRS2*	3	*f2*	*N1*	*C1*	*LRI2*	*LRS2*	3	*f2*	*N1*	*C1*	*LRI3*	*LRS2*
4	*f2*	*N2*	*C1*	*LRI1*	*LRS1*	4	*f2*	*N2*	*C1*	*LRI2*	*LRS1*	4	*f2*	*N2*	*C1*	*LRI3*	*LRS1*
4	*f2*	*N2*	*C1*	*LRI1*	*LRS2*	4	*f2*	*N2*	*C1*	*LRI2*	*LRS2*	4	*f2*	*N2*	*C1*	*LRI3*	*LRS2*
5	*f1*	*N1*	*C2*	*LRI1*	*LRS1*	5	*f1*	*N1*	*C2*	*LRI2*	*LRS1*	5	*f1*	*N1*	*C2*	*LRI3*	*LRS1*
5	*f1*	*N1*	*C2*	*LRI1*	*LRS2*	5	*f1*	*N1*	*C2*	*LRI2*	*LRS2*	5	*f1*	*N1*	*C2*	*LRI3*	*LRS2*
6	*f1*	*N2*	*C2*	*LRI1*	*LRS1*	6	*f1*	*N2*	*C2*	*LRI2*	*LRS1*	6	*f1*	*N2*	*C2*	*LRI3*	*LRS1*
6	*f1*	*N2*	*C2*	*LRI1*	*LRS2*	6	*f1*	*N2*	*C2*	*LRI2*	*LRS2*	6	*f1*	*N2*	*C2*	*LRI3*	*LRS2*
7	*f2*	*N1*	*C2*	*LRI1*	*LRS1*	7	*f2*	*N1*	*C2*	*LRI2*	*LRS1*	7	*f2*	*N1*	*C2*	*LRI3*	*LRS1*
7	*f2*	*N1*	*C2*	*LRI1*	*LRS2*	7	*f2*	*N1*	*C2*	*LRI2*	*LRS2*	7	*f2*	*N1*	*C2*	*LRI3*	*LRS2*
8	*f2*	*N2*	*C2*	*LRI1*	*LRS1*	8	*f2*	*N2*	*C2*	*LRI2*	*LRS1*	8	*f2*	*N2*	*C2*	*LRI3*	*LRS1*
8	*f2*	*N2*	*C2*	*LRI1*	*LRS2*	8	*f2*	*N2*	*C2*	*LRI2*	*LRS2*	8	*f2*	*N2*	*C2*	*LRI3*	*LRS2*

* f (mm/rev); ** N (rpm).

**Table 3:** Chemical composition of the materials used for the manufacturing specimens.

UNS M11917 (AZ91D)	UNS A92024 (AA2024 T351)
Al 8.30–9.70%	Al 90.7–94.7%
Cu ≤ 0.03%	Cr ≤ 0.1%
Fe ≤ 0.005%	Cu 3.8–4.9%
Mg 90%	Fe ≤ 0.5%
Mn ≥ 0.13%	Mg 1.2–1.8%
Ni ≤ 0.002%	Mn 0.3–0.9%
Si ≤ 0.1%	Si ≤ 0.5%
Zn 0.35–1%	Ti ≤ 0.15%
–	Zn ≤ 0.25%

**Table 4:** Factors, levels, and values. CCA—cold compressed air.

Factors	Level Values
Feed rate, *f* (mm/rev)	*f1* = 0.05; *f2* = 0.10
Spindle speed, *N* (rpm)	*N1* = 500; *N2* = 1200
Type of cooling system, *C*	*C1* = CCA; *C2* = dry
Location relative to the insert, *LRI*	*LRI1* = Mg; *LRI2* = Al; *LRI3* = Mg
Location relative to the specimen, *LRS*	*LRS1* = specimen entry zone; *LRS2* = specimen exit zone

**Table 5:** The arithmetical mean roughness (*Ra*) and the average maximum height (*Rz*) obtained during the measurement tests.

No.	*f* (mm/rev)	*N* (rpm)	*C*	*LRI*	*LRS*	*Ra* (µm)	*Rz* (µm)
1	0.05	500	*CCA*	*LRI1*	*LRS1*	0.35	2.70
1	0.05	500	*CCA*	*LRI1*	*LRS2*	0.81	4.30
2	0.05	1200	*CCA*	*LRI1*	*LRS1*	1.06	6.10
2	0.05	1200	*CCA*	*LRI1*	*LRS2*	1.23	7.20
3	0.10	500	*CCA*	*LRI1*	*LRS1*	1.27	8.50
3	0.10	500	*CCA*	*LRI1*	*LRS2*	1.31	7.00
4	0.10	1200	*CCA*	*LRI1*	*LRS1*	1.03	6.40
4	0.10	1200	*CCA*	*LRI1*	*LRS2*	1.33	7.20
5	0.05	500	*Dry*	*LRI1*	*LRS1*	1.22	6.90
5	0.05	500	*Dry*	*LRI1*	*LRS2*	0.19	2.20
6	0.05	1200	*Dry*	*LRI1*	*LRS1*	0.61	4.60
6	0.05	1200	*Dry*	*LRI1*	*LRS2*	0.73	5.70
7	0.10	500	*Dry*	*LRI1*	*LRS1*	0.73	5.40
7	0.10	500	*Dry*	*LRI1*	*LRS2*	0.48	3.60
8	0.10	1200	*Dry*	*LRI1*	*LRS1*	0.98	7.00
8	0.10	1200	*Dry*	*LRI1*	*LRS2*	0.62	4.00
1	0.05	500	*CCA*	*LRI2*	*LRS1*	2.94	16.80
1	0.05	500	*CCA*	*LRI2*	*LRS2*	3.00	16.20
2	0.05	1200	*CCA*	*LRI2*	*LRS1*	1.31	7.20
2	0.05	1200	*CCA*	*LRI2*	*LRS2*	3.95	18.00
3	0.10	500	*CCA*	*LRI2*	*LRS1*	1.20	8.70
3	0.10	500	*CCA*	*LRI2*	*LRS2*	2.11	11.50
4	0.10	1200	*CCA*	*LRI2*	*LRS1*	0.13	1.50
4	0.10	1200	*CCA*	*LRI2*	*LRS2*	2.91	15.20
5	0.05	500	*Dry*	*LRI2*	*LRS1*	1.24	6.80
5	0.05	500	*Dry*	*LRI2*	*LRS2*	1.30	6.80
6	0.05	1200	*Dry*	*LRI2*	*LRS1*	1.88	11.20
6	0.05	1200	*Dry*	*LRI2*	*LRS2*	0.51	4.00
7	0.10	500	*Dry*	*LRI2*	*LRS1*	0.87	5.10
7	0.10	500	*Dry*	*LRI2*	*LRS2*	0.33	2.40
8	0.10	1200	*Dry*	*LRI2*	*LRS1*	1.66	8.40
8	0.10	1200	*Dry*	*LRI2*	*LRS2*	1.70	9.80
1	0.05	500	*CCA*	*LRI3*	*LRS1*	0.48	4.00
1	0.05	500	*CCA*	*LRI3*	*LRS2*	0.59	3.40
2	0.05	1200	*CCA*	*LRI3*	*LRS1*	0.64	4.00
2	0.05	1200	*CCA*	*LRI3*	*LRS2*	0.56	3.50
3	0.10	500	*CCA*	*LRI3*	*LRS1*	0.56	3.20
3	0.10	500	*CCA*	*LRI3*	*LRS2*	0.78	5.10
4	0.10	1200	*CCA*	*LRI3*	*LRS1*	1.13	6.10
4	0.10	1200	*CCA*	*LRI3*	*LRS2*	0.92	5.90
5	0.05	500	*Dry*	*LRI3*	*LRS1*	0.34	2.40
5	0.05	500	*Dry*	*LRI3*	*LRS2*	0.67	6.50
6	0.05	1200	*Dry*	*LRI3*	*LRS1*	0.53	3.50
6	0.05	1200	*Dry*	*LRI3*	*LRS2*	0.63	3.80
7	0.10	500	*Dry*	*LRI3*	*LRS1*	0.46	2.80
7	0.10	500	*Dry*	*LRI3*	*LRS2*	0.42	3.60
8	0.10	1200	*Dry*	*LRI3*	*LRS1*	0.59	3.80
8	0.10	1200	*Dry*	*LRI3*	*LRS2*	0.53	3.90

**Table 6:** Values of *Ra* in each plate at the entry and at the exit zones of the holes.

No.	*F (mm/rev)*	*N (rpm)*	*C*	*Ra* (µm)					
				*LRI1*		*LRI2*		*LRI3*	
				*LRS1*	*LRS2*	*LRS1*	*LRS2*	*LRS1*	*LRS2*
1	*0.05*	*500*	*CCA*	0.35	0.81	2.94	3.00	0.48	0.59
2	*0.05*	*1200*	*CCA*	1.06	1.23	1.31	3.95	0.64	0.56
3	*0.10*	*500*	*CCA*	1.27	1.31	1.20	2.11	0.56	0.78
4	*0.10*	*1200*	*CCA*	1.03	1.33	0.13	2.91	1.13	0.92
5	*0.05*	*500*	*Dry*	1.22	0.19	1.24	1.30	0.34	0.67
6	*0.05*	*1200*	*Dry*	0.61	0.73	1.88	0.51	0.53	0.63
7	*0.10*	*500*	*Dry*	0.73	0.48	0.87	0.33	0.46	0.42
8	*0.10*	*1200*	*Dry*	0.98	0.62	1.66	1.70	0.59	0.53

**Table 7:** Values of *Rz* in each plate at the entry and exit zones of the holes.

No.	*F (mm/rev)*	*N (rpm)*	*C*	*Rz* (µm)					
				*LRI1*		*LRI2*		*LRI3*	
				*LRS1*	*LRS2*	*LRS1*	*LRS2*	*LRS1*	*LRS2*
1	*0.05*	*500*	*CCA*	2.70	4.30	16.80	16.20	4.00	3.40
2	*0.05*	*1200*	*CCA*	6.10	7.20	7.20	18.00	4.00	3.50
3	*0.10*	*500*	*CCA*	8.50	7.00	8.70	11.50	3.20	5.10
4	*0.10*	*1200*	*CCA*	6.40	7.20	1.50	15.20	6.10	5.90
5	*0.05*	*500*	*Dry*	6.90	2.20	6.80	6.80	2.40	6.50
6	*0.05*	*1200*	*Dry*	4.60	5.70	11.20	4.00	3.50	3.80
7	*0.10*	*500*	*Dry*	5.40	3.60	5.10	2.40	2.80	3.60
8	*0.10*	*1200*	*Dry*	7.00	4.00	8.40	9.80	3.80	3.90

**Table 8:** Homogeneity test of variances for factors *f* and *N* and response variables *LnRa* and *LnRz*.

	*f* (mm/rev)		*N* (rpm)	
	Levene Statistic	Significance	Levene Statistic	Significance
*LnRa*	0.30	0.59	0.58	0.46
*LnRz*	0.21	0.65	0.13	0.72

**Table 9:** Outcome of the first iteration for the ANOVA over Ln*Ra*.

Source	DF *	Sum of Squares	Mean Square	F-value	*p* > F
Corrected Model	23	13.037	0.567	1.415	0.202
Intercept	1	1.486	1.486	3.709	0.066
*LRI*	2	4.983	2.491	6.217	0.007
*N*	1	0.306	0.306	0.764	0.391
*f*	1	0.010	0.010	0.025	0.875
*C*	1	1.882	1.882	4.697	0.040
*LRI* × *N*	2	0.365	0.183	0.456	0.639
*LRI* × *f*	2	1.766	0.883	2.203	0.132
*LRI* × *C*	2	0.070	0.035	0.087	0.917
*N* × *f*	1	0.002	0.002	0.006	0.938
*N* × *C*	1	0.306	0.306	0.764	0.391
*f* × *C*	1	0.003	0.003	0.008	0.929
*LRI* × *N* × *f*	2	0.368	0.184	0.459	0.638
*LRI* × *N* × *C*	2	0.801	0.401	1.000	0.383
*LRI* × *f* × *C*	2	0.906	0.453	1.130	0.340
*N* × *f* × *C*	1	0.576	0.576	1.437	0.242
*LRI* × *N* × *f* × *C*	2	0.694	0.347	0.865	0.434
Error	24	9.617	0.401	–	–
Total	48	24.141	–	–	–
Corrected Total	47	22.654	–	–	–

* DF, degrees of freedom.

**Table 10:** Outcome of the last iteration for the ANOVA over Ln*Ra*.

Source	DF *	Sum of Squares	Mean Square	F-value	*p* > F
Corrected Model	5	6.935a	1.387	3.706	0.007
Intercept	1	1.486	1.486	3.971	0.053
*LRI*	2	4.983	2.491	6.656	0.003
*C*	1	1.882	1.882	5.029	0.030
Error	42	15.720	0.374	–	–
Total	48	24.141	–	–	–
Corrected Total	47	22.654	–	–	–

* DF, degrees of freedom.

**Table 11:** Outcome of the first iteration for the ANOVA over Ln*Rz*.

Source	DF *	Sum of Squares	Mean Square	F-value	*p* > F
Corrected Model	23	8.726a	0.379	1.600	0.130
Intercept	1	137.220	137.220	578.50	0.000
*LRI*	2	3.673	1.836	7.742	0.003
*C*	1	0.963	0.963	4.061	0.055
*f*	1	0.009	0.009	0.039	0.845
*N*	1	0.150	0.150	0.633	0.434
*LRI* × *C*	2	0.190	0.095	0.402	0.674
*LRI* × *f*	2	1.027	0.514	2.165	0.137
*LRI* × *N*	2	0.172	0.086	0.363	0.699
*C* × *f*	1	0.014	0.014	0.057	0.813
*C* × *N*	1	0.232	0.232	0.978	0.332
*f* × *N*	1	0.011	0.011	0.048	0.828
*LRI* × *C* × *f*	2	0.490	0.245	1.034	0.371
*LRI* × *C* × *N*	2	0.840	0.420	1.770	0.192
*LRI* × *f* × *N*	2	0.381	0.190	0.803	0.460
*C* × *f* × *N*	1	0.311	0.311	1.310	0.264
*LRI* × *C* × *f* × *N*	2	0.263	0.131	0.553	0.582
Error	24	5.693	0.237		
Total	48	151.639			
Corrected Total	47	14.419			

* DF, degrees of freedom.

**Table 12:** Outcome of the last iteration for the ANOVA over Ln*Rz*.

Source	DF *	Sum of Squares	Mean Square	F-value	*p* > F
Corrected Model	5	4.826	0.965	4.226	0.003
Intercept	1	137.22	137.220	600.78	0.000
*LRI*	2	3.673	1.836	8.040	0.001
*C*	1	0.963	0.963	4.217	0.046
Error	42	9.593	0.228		
Total	48	151.63			
Corrected Total	47	14.419			

* DF, degrees of freedom.

**Table 13:** Percentage variability of the statistically significant effects obtained from ANOVA.

Source	*Ra*		*Rz*	
	Sum of Squares	Variability Percentage	Sum of Squares	Variability Percentage
*LRI*	5.0	72.6%	3.7	79.2%
*C*	1.9	27.4%	1.0	20.8%
Total	6.9	100%	4.6	100%
